# Lipid changes during the perioperative period in patients with early breast cancer: a real-world retrospective analysis

**DOI:** 10.1186/s12893-021-01396-9

**Published:** 2021-11-12

**Authors:** Tao He, Zhu Wang, Yunhao Wu, Xinyi Zhang, Xu Li, Jiayuan Li, Liang Du, Jie Chen, Qing Lv

**Affiliations:** 1 Department of Breast Surgery, West China School of Medicine/West China Hospital, Sichuan University, Chengdu, 610041 Sichuan China; 2 Laboratory of Molecular Diagnosis of Cancer, West China Hospital, Sichuan University, Chengdu, 610041 Sichuan China; 3 West China School of Medicine/West China Hospital, Sichuan University, Chengdu, 610041 Sichuan China; 4 Center of Biostatistics, Design, Measurement and Evaluation (CBDME), Department of Clinical Research Management, West China Hospital, Sichuan University, Chengdu, 610041 Sichuan China; 5 West China School of Public Health and West China Fourth Hospital, Sichuan University, Chengdu, 610041 Sichuan China; 6 Chinese Evidence-Based Medicine Center, West China Hospital, Sichuan University, Chengdu, 610041 Sichuan China; 7 Department of Breast Surgery, West China Hospital, Sichuan University, Chengdu, 610041 Sichuan China

**Keywords:** Breast cancer, Surgery, Dyslipidemia

## Abstract

**Background:**

Surgery remains the major treatment for early breast cancer (BC), but surgery itself is also a trauma which might induce alterations in lipid metabolism. The aim of this study was to investigate the changes in lipid profiles and to explore factors associated with lipid changes pre- and postoperation.

**Methods:**

We retrospectively analyzed the pre- and postoperative serum lipid profiles of 1934 BC patients.

**Results:**

The levels of triglycerides (TG) (*p* < 0.001) and low-density lipoprotein cholesterol (LDL) (*p* < 0.001) were significantly elevated after surgery, while the levels of high-density lipoprotein cholesterol (HDL) (*p* < 0.001) were significantly decreased. After surgery, 27.76% of patients with preoperative ortholiposis developed dyslipidemia. Postmenopausal BC patients had a higher incidence of dyslipidemia (32.31%) after surgery than premenopausal BC patients (26.07%; *p* = 0.041). Additionally, patients with BMI > 24 (34.92%) had a higher incidence of dyslipidemia than patients with BMI ≤ 24 (24.84%; *p* = 0.001). Moreover, the magnitudes of the TG increase (p < 0.001), cholesterol (TC) increase (p = 0.013) and LDL increase (p = 0.015) in the premenopausal group were all greater than those in the postmenopausal group. After adjusting for multiple baseline covariates, preoperative hyperlipidemia and progesterone receptor (PR)-positive status were significantly associated with elevated TG, TC and LDL levels after surgery.

**Conclusions:**

Serum lipid profiles of BC patients may increase after surgery, especially premenopausal patients. Additionally, postmenopausal and overweight patients may have a higher risk of being diagnosed with dyslipidemia after surgery. Therefore, lipid monitoring, dyslipidemia prevention and corresponding interventions should be taken into consideration during the perioperative period.

## Introduction

Breast cancer is one of the most prevalent malignancies worldwide [1]. According to the estimates from the Global Cancer Statistics, there were 2,088,849 newly diagnosed breast cancer cases in 2018, accounting for 24.2% of cancer cases among females [2]. In terms of mortality, breast cancer, accounting for 15% of cancer deaths among females, is the leading cause of female cancer-related deaths [3].

Currently, complete surgery remains the major curative treatment for localized breast cancer [4]. However, surgery itself is also a trauma that might induce the stress response of patients to surgery [5]. There are several systemic responses caused by surgical trauma, including endocrinological, immunological, hematological and psychological effects [6]. Recent studies have shown that carbohydrate, protein, fat, water and electrolyte metabolism might all be affected by the stress response to surgery [5, 7]. Among these changes, fat metabolism and serum lipid profile variations are one of the most important and obvious indicators.

Recently, some studies have revealed that dyslipidemia is associated with metastasis in patients with BC [7], and the regular oral administration of statins attenuates the spread of breast cancer metastases [8]. Additionally, dyslipidemia was an independent risk factor for inducing cardiovascular disease (CVD) [9]. A scientific statement from the American Heart Association revealed that CVD and breast cancer have several overlapping risk factors. Specifically, for older women, CVD poses a greater mortality threat than breast cancer itself [10]. As such, it is vital to monitor the serum lipid profiles of patients with BC after surgery in the clinic.

However, few studies have explored the serum lipid variations associated with the surgical stress response until now. Thus, we retrospectively investigated the serum lipid levels pre- and postoperation and explored the influencing factors of serum lipid changes.

## Methods and materials

### Patient selection

The medical records of all consecutive patients diagnosed with early breast cancer were retrospectively collected from the Department of Breast Surgery, West China Hospital, Sichuan University, between February 2009 and December 2016. The Institutional Review Board and Ethics Committee of West China Hospital approved this retrospective study (IRB No. 2016L03115). Data were accessed anonymously, waiving the requirement for consent from study participants. All methods were carried out in accordance with the relevant guidelines and regulations. The inclusion criteria were as follows: (1) female patients aged ≥ 18 years; (2) patients surgically treated and pathologically diagnosed with breast cancer; and (3) patients who had adequate organ function with Eastern Cooperative Oncology Group (ECOG) ≤ 2. The exclusion criteria were as follows: (1) patients with BC and other malignancies; (2) patients with distant metastatic breast cancer; (3) patients who previously underwent other operations; (4) patients who received neoadjuvant treatment; (5) patients taking drugs affecting lipid levels; (6) pregnant and lactating women; and (7) patients with incomplete data.

### Data collection and evaluated parameters

Clinicopathological data were reviewed from medical records. The variables of interest included height, weight, body mass index (BMI), age at diagnosis, menopausal status, smoking status, tumor location, surgical procedures, Tumor Node Metastasis (TNM) stage [11], ER status, PR status, preoperative hypertension, preoperative hyperlipidemia and pre- and postoperative lipid profile values (blood samples were collected in the morning on an empty stomach within a week before surgery and within 2 weeks after surgery). According to the Chinese guidelines for the management of dyslipidemia, dyslipidemia was considered if patients met at least one of the following criteria: TG ≥ 1.7 mmol/L, TC ≥ 5.2 mmol/L, LDL ≥ 3.4 mmol/L, and HDL ≤ 1.0 mmol/L [12]. Body weight and height were assessed for the determination of BMI, which was calculated as the weight divided by the height squared (kg/m^2^). Patients were classified as postmenopausal if they were not pregnant, were aged over 40 years and had no menstruation for at least 12 months [13]. Since menopausal status is closely related to serum lipids, the participants in this study were further stratified into two groups by menopausal status: premenopausal and postmenopausal. Additionally, BMI may influence serum lipids [14], so we then stratified the participants into two groups: BMI ≤ 24 and BMI > 24.

### Statistical analysis

All statistical analyses were performed using the Statistical Package for the Social Sciences (SPSS), version 22.0 for Windows (SPSS Inc., Chicago, IL, USA). Quantitative results are expressed as the mean ± standard deviation (SD). A two-sided *p*-value of less than 0.05 was considered statistically significant. A paired-sample T test was used to compare the lipid values pre- and postoperation. One-way analysis of variance was used to assess significant differences in the magnitude of variations in lipid profiles pre- and postoperation between the premenopausal and postmenopausal groups and between the BMI ≤ 24 and BMI > 24 groups. The magnitudes of variation in lipid profiles pre- and postoperation were calculated, and the difference was calculated as the postoperative lipid values minus the preoperative lipid values. The Chi-square test was used to calculate the incidence of postoperative hyperlipidemia in patients with normal serum lipids before surgery. Moreover, we incorporated the significant variables associated with the increase in serum lipid profiles after surgery, which were identified by univariate analysis (*p* < 0.05), into a logistic regression model to identify the independent factors that were correlated with the elevated serum lipid profiles.

## Results

### Patient characteristics

A total of 1934 eligible and consecutive patients with BC were identified for inclusion in this retrospective cohort study. The mean age of the entire cohort was 48.90 ± 5.38 years, with a median age of 48 years old. The baseline characteristics of the patients were summarized in Table 1. Before surgery, all patients were assessed by “West China mood index” scale and their scores were higher than normal. Additionally, all patients examined blood routine examination, blood biochemistry, mammary color ultrasound and breast X-ray before surgery. All patients received general anesthesia and there was no severe adverse reaction after surgery.

**Table 1:** Demographic and clinical characteristics of the study population (n = 1934)

	Premenopause (N = 1184)	Postmenopause (N = 750)	p
Age, mean (SD), y	43.22 (5.65)	57.86 (6.73)	*p* < *0.01*
BMI (kg/m^2^), n			*p* < *0.01*
≤ 24	824	441	
> 24	360	309	
Smoking, n			*p* = 0.204
Yes	15	5	
No	1169	745	
Preoperative hypertension, n			*p* < *0.01*
Yes	41	68	
No	1143	682	
Preoperative hyperlipidemia, n			*p* < *0.01*
Yes	382	452	
No	802	298	
Tumor location, n			*p* = 0.651
Left breast	619	400	
Right breast	565	350	
TNM stage, n			*p* = 0.388
Stage I	417	253	
Stage II	743	487	
Stage III	24	10	
Surgical procedures, n			*p* < *0.01*
Mastectomy	985	713	
Breast conserving operation	146	31	
Breast reconstruction	53	6	
Molecular subtyping, n			*p* < *0.01*
LuminaA	73	45	
LuminaB	160	173	
Triple negative	139	77	
Her-2 positive	812	455	

*SD* standard deviation; *BMI* body mass index

### Serum lipid alterations after surgery

**Fig. 1:**
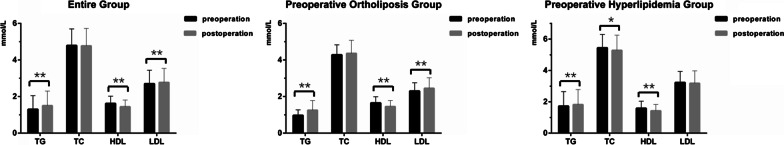
Changes in serum lipid profiles between pre- and post-operation in the entire group, pre-operation ortholiposis group and pre-operation hyperlipidemia group, respectively. **p < 0.001 for comparison of pre- vs post-operation

**Fig. 2:**
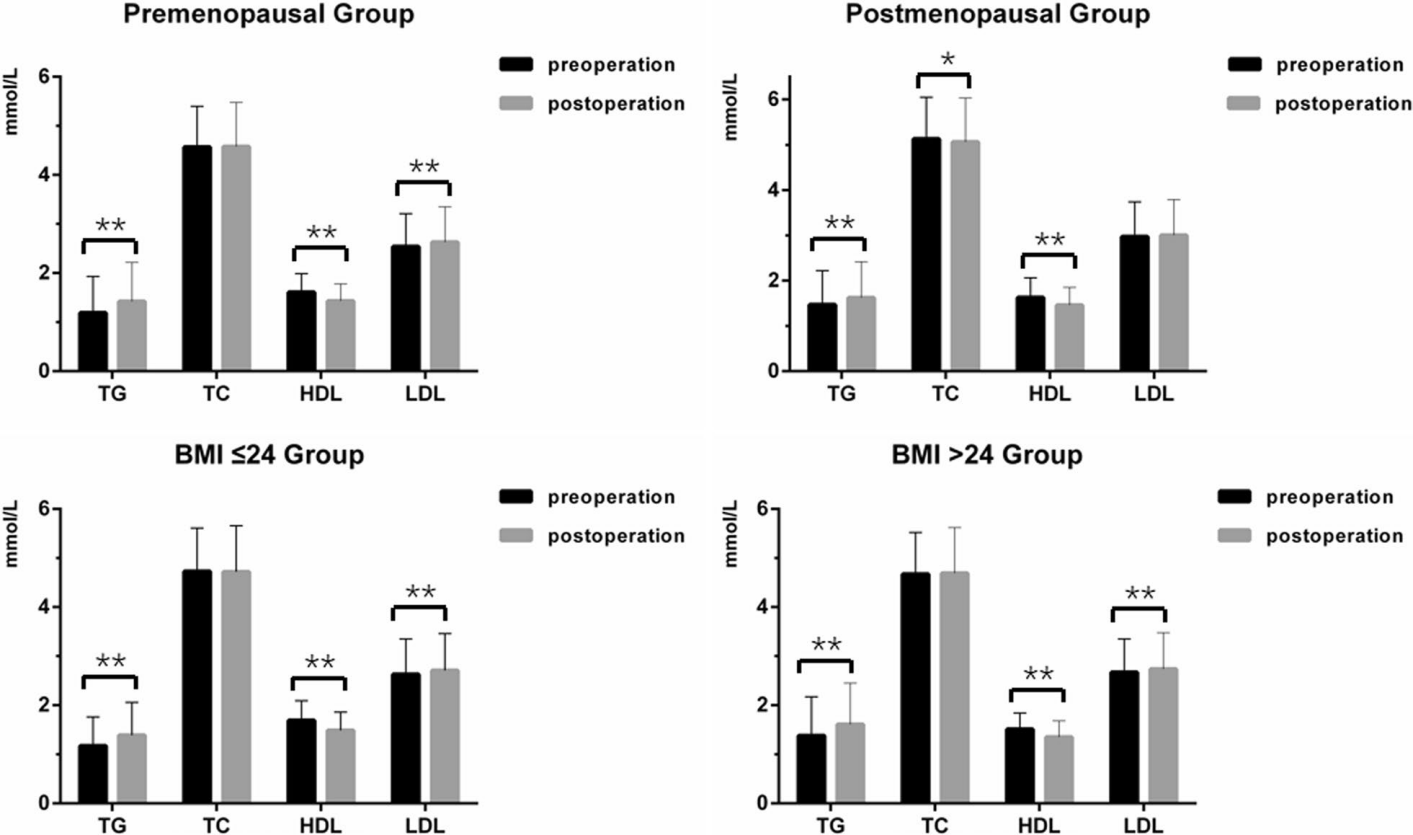
Changes in serum lipid profiles between pre- and post-operation in the premenopause group, postmenopause group, BMI ≤ 24 group and BMI > 24 group, respectively. *p < 0.005 for comparison of pre- vs post-operation. **p < 0.001 for comparison of pre- vs post-operation

The lipid level variations after surgery are shown in Table 2, Figs. 1 and 2. In this study, there were 1151 patients with elevated TG levels, 785 patients with elevated TC levels, 1009 patients with elevated LDL levels and 1548 patients with decreased HDL levels after surgery. For the entire group, we found a significant increase in TG (*p* < 0.001) and LDL (*p* < 0.001), while a marked decrease in HDL (*p* < 0.001) was observed postoperatively. However, in terms of TC, there was no significant difference between pre- and postoperation (*p* = 0.227).

**Table 2:** Comparison of the lipid profiles between pre- and post-operation

Parameters	Pre-operation	Post-operation	*p*
Entire group (N = 1934)			
TG	1.30 ± 0.75	1.50 ± 0.80	< 0.001
TC	4.79 ± 0.91	4.77 ± 0.96	0.227
HDL	1.62 ± 0.40	1.44 ± 0.37	< 0.001
LDL	2.70 ± 0.74	2.77 ± 0.77	< 0.001
Preoperative ortholiposis (N = 1088)			
TG	0.97 ± 0.30	1.25 ± 0.53	< 0.001
TC	4.28 ± 0.55	4.36 ± 0.72	< 0.001
HDL	1.65 ± 0.34	1.45 ± 0.33	< 0.001
LDL	2.30 ± 0.45	2.45 ± 0.58	< 0.001
Preoperative hyperlipidemia (N = 846)			
TG	1.72 ± 0.93	1.82 ± 0.96	< 0.001
TC	5.44 ± 0.86	5.28 ± 0.97	< 0.001
HDL	1.58 ± 0.46	1.42 ± 0.41	< 0.001
LDL	3.23 ± 0.71	3.18 ± 0.79	< 0.001
Premenopause (N = 1184)			
TG	1.19 ± 0.74	1.42 ± 0.80	< 0.001
TC	4.57 ± 0.83	4.58 ± 0.90	0.545
HDL	1.61 ± 0.38	1.43 ± 0.35	< 0.001
LDL	2.54 ± 0.67	2.63 ± 0.72	< 0.001
Postmenopause (N = 750)			
TG	1.47 ± 0.75	1.62 ± 0.80	< 0.001
TC	5.13 ± 0.92	5.06 ± 0.97	0.007
HDL	1.63 ± 0.43	1.46 ± 0.39	< 0.001
LDL	2.97 ± 0.77	3.00 ± 0.79	0.186
BMI ≤ 24 (N = 1265)			
TG	1.17 ± 0.59	1.39 ± 0.67	< 0.001
TC	4.73 ± 0.88	4.72 ± 0.94	0.573
HDL	1.69 ± 0.40	1.49 ± 0.37	< 0.001
LDL	2.63 ± 0.72	2.71 ± 0.75	< 0.001
BMI > 24 (N = 669)			
TG	1.38 ± 0.79	1.61 ± 0.84	< 0.001
TC	4.67 ± 0.85	4.69 ± 0.93	0.338
HDL	1.51 ± 0.33	1.35 ± 0.33	< 0.001
LDL	2.67 ± 0.68	2.74 ± 0.74	< 0.001

*TG* triglycerides, *TC* total cholesterols, *HDL* high-density lipoprotein, *LDL* low-density lipoprotein, *BMI* body mass index

In addition, the participants were then stratified into two groups by preoperative lipid status: preoperative ortholiposis and preoperative hyperlipidemia. For the preoperative ortholiposis group, we found an increase in TG, TC and LDL levels (*p* < 0.001) and a decrease in HDL levels after surgery (*p* < 0.01). For the preoperative hyperlipidemia group, an increase was also noted in the levels of TG and TC between pre- and postoperation (*p* < 0.001), and a decrease was found in both LDL and HDL (*p* < 0.001) after surgery.

Since menopausal status is closely related to serum lipid profiles, the study participants were stratified into two groups by menopausal status: premenopausal and postmenopausal groups. For the premenopausal group, we found an increase in TG and LDL levels (*p* < 0.001) and a decrease in HDL levels after surgery (*p* < 0.01), while there was no significant difference in TC levels between pre- and postoperation (*p* = 0.545). For the postmenopausal group, an increase was also noted in the levels of TG between pre- and postoperation (*p* < 0.001); a decrease was found in TC (*p* = 0.007) and HDL (*p* < 0.001) after surgery.

Additionally, we stratified the participants into two groups by BMI: BMI ≤ 24 and BMI > 24. We found an increase in TG, TC and LDL levels (p < 0.001) and a decrease in HDL levels after surgery (p < 0.01) in both groups.

### Incidence of postoperative hyperlipidemia in patients with preoperative ortholiposis

**Fig. 3:**
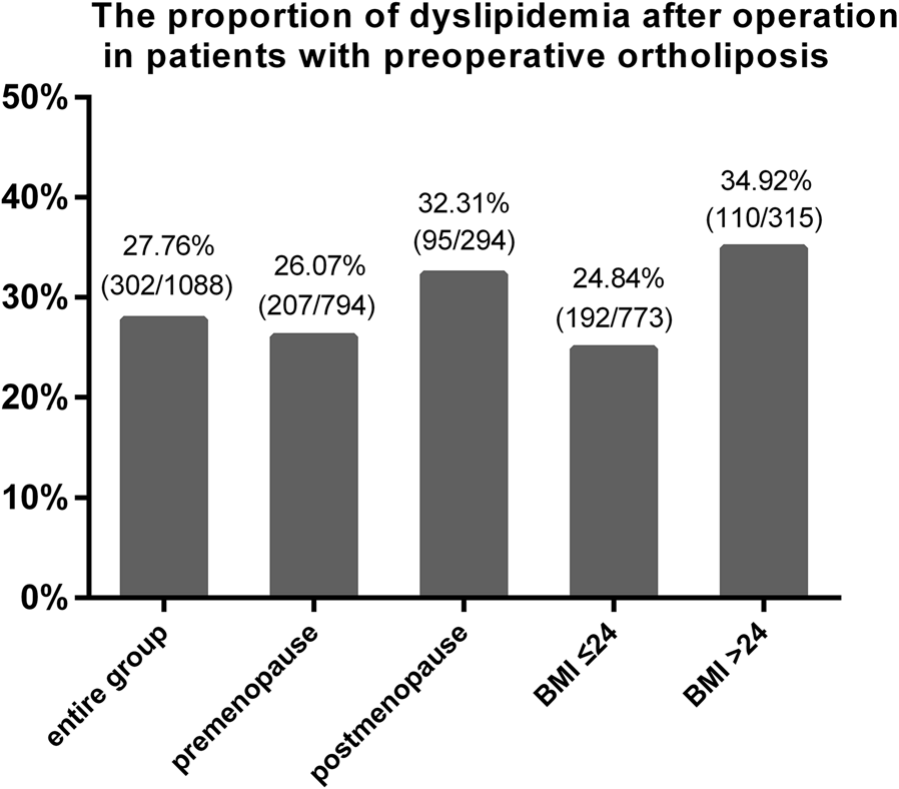
The percentage of dyslipidaemia after operation in patients with pre-operation ortholiposis

Among the 1934 patients, 1088 patients had normal serum lipid profiles before surgery. As many patients had increased serum lipid levels after surgery, we further assessed the percentage of patients newly diagnosed with postoperative dyslipidemia (Fig. 3). Among the 1088 patients with preoperative ortholiposis, 302 patients (27.76%) were newly diagnosed with dyslipidemia after surgery. Stratified by menopausal status, the incidence of newly diagnosed dyslipidemia was higher in the postmenopausal group (32.31%) than in the premenopausal (26.07%) group (*p* = 0.041). Then, stratified by BMI status, we found that the incidence of newly diagnosed dyslipidemia was higher in patients with BMI > 24 (34.92%) than in patients with BMI ≤ 24 (24.84%; *p* = 0.001).

### Comparison of the magnitude of lipid alterations after surgery

**Fig. 4:**
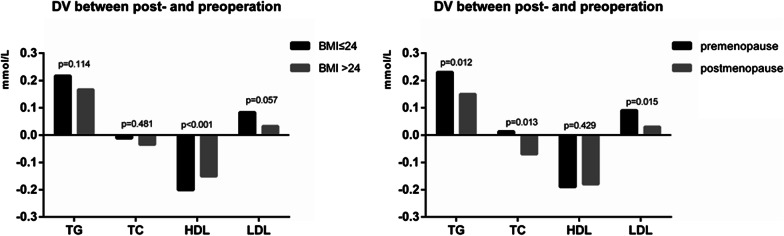
Difference value of serum lipid levels between post-and pre-operation in BMI ≤ 24 group and BMI > 24 group; pre-and post-menopausal group, respectively (difference value = lipid value of post-operation minus lipid value of pre-operation)

To explore the effects of menopausal status and BMI status on serum lipid level variations, we carried out further analyses. The data is provided in Table 3 and Fig. 4. The levels of TG increased after surgery in both the premenopausal and postmenopausal groups (*p* < 0.001), and the magnitude of the TG increase after surgery in the premenopausal group was greater than that in the postmenopausal group (*p* = 0.012). For TC, the variations in TC pre- and postoperation were significantly different between the premenopausal and postmenopausal groups, and the elevation in TC was greater in the premenopausal group (*p* = 0.013). For HDL, we found a decrease in both the premenopausal and postmenopausal groups (*p* < 0.001), but there was no significant difference in the magnitude of variation in HDL between the groups (*p* = 0.429). For LDL, we found an increase in the premenopausal group (*p* < 0.001), and the magnitude of LDL increase was greater in the premenopausal group than in the postmenopausal group (*p* = 0.015).

**Table 3:** Comparison of change values of blood lipid between pre- and postoperation

Parameters	Premenopause (N = 1184)	Postmenopause (N = 750)	*p*
TG	0.23 ± 0.69	0.15 ± 0.63	0.012
TC	0.013 ± 0.71	− 0.07 ± 0.72	0.013
HDL	− 0.19 ± 0.29	− 0.18 ± 0.30	0.429
LDL	0.09 ± 0.53	0.03 ± 0.56	0.015

Parameters	BMI ≤ 24 (N = 1265)	BMI > 24 (N = 669)	*p*
TG	0.22 ± 0.57	0.17 ± 0.81	< 0.001
TC	− 0.01 ± 0.57	− 0.04 ± 0.72	0.689
HDL	− 0.20 ± 0.30	− 0.15 ± 0.27	< 0.001
LDL	0.08 ± 0.53	0.03 ± 0.58	0.812

*TG* triglycerides, *TC* total cholesterols, *HDL* high-density lipoprotein, *LDL* low-density lipoprotein

Additionally, we compared the magnitude of lipid alterations after surgery between patients with BMI > 24 and BMI ≤ 24. The levels of HDL decreased after surgery in both the BMI > 24 and BMI ≤ 24 groups, and the magnitude of decreased HDL was greater in the BMI ≤ 24 group (*p* < 0.001). However, there was no significant difference in the magnitude of variation in TG (*p* = 0.114), TC (*p* = 0.481) and LDL (*p* = 0.057).

### Factors related to the increase in lipid profiles after surgery

To explore the potential clinicopathological parameters associated with increased lipid profiles after surgery, we performed univariate and multivariate analyses (Table 4). With respect to TG, TC and LDL levels, multivariate analysis showed that PR-positive status and preoperative hyperlipidemia were associated with elevated TG, TC and LDL levels after surgery (*p* < 0.05). In terms of HDL, both univariate and multivariate analyses showed that none of the factors were associated with decreased HDL levels after surgery.

**Table 4:** Factors identified to be associated with the increase in lipid profiles based on multivariate analysis, by stepwise logistic regression

	Univariate analysis			Multivariate analysis		
	OR	95% CI for OR	p	OR	95% CI for OR	p
TG: increase (n = 1151) vs not increase (n = 783)						
Age						
≤ 40	1					
40–50	1.067	0.825–1.381	0.622			
> 50	0.712	0.548–0.926	0.011			
Premenopause						
Pre-	1					
Post-	0.671	0.557–0.808	< 0.001			
ER						
ER−	1					
ER+	1.570	1.293–1.905	< 0.001			
PR						
PR−	1			1		
PR+	1.656	1.371–2.000	< 0.001	1.411	1.046–1.904	0.024
Preoperative hyperlipidemia	2.212	1.838–2.663	< 0.001	2.121	1.745–2.578	< 0.001
TC: increase (n = 785) vs no increase (n = 1149)						
Premenopause						
Pre-	1					
Post-	0.801	0.664–0.966	0.02			
ER						
ER−	1					
ER+	1.253	1.029–1.526	0.025			
PR						
PR−	1			1		
PR+	1.409	1.163–1.707	< 0.001	1.479	1.093–2.003	0.011
Preoperative hyperlipidemia	1.710	1.419–2.061	< 0.001	1.672	1.376–2.030	< 0.001
LDL: increase (n = 1009) vs not increase (n = 925)						
Premenopause						
Pre-	1					
Post-	0.813	0.676–0.976	0.027			
ER						
ER−	1					
ER+	1.349	1.112–1.635	0.002			
PR						
PR−	1			1		
PR+	1.443	1.196–1.740	< 0.001	1.407	1.164–1.701	< 0.001
Preoperative hyperlipidemia	1.863	1.552–2.237	< 0.001	1.838	1.530–2.209	< 0.001

*TG* triglycerides, *TC* total cholesterols, *LDL* low-density lipoprotein, *ER* estrogen receptor, *PR* progesterone receptor

## Discussion

In this study, we focused on the variations in serum lipid profiles pre- and postoperation in patients with BC. To our knowledge, this is one of the studies that contains a relatively large sample size to investigate lipid changes after breast cancer surgery. In this entire cohort, we found that serum lipid profiles of BC patients may increase after surgery, especially premenopausal patients. Additionally, postmenopausal and overweight patients may have a higher risk of being diagnosed with dyslipidemia after surgery.

Previously, a few studies have addressed the stress response to trauma and surgery [5]. The activation of the hypothalamic–pituitary–adrenal (HPA) axis and the sympathetic nervous system (SNS) are characteristics of the stress response to surgical trauma [15]. With the activation of the HPA axis and SNS, several hormones, such as adrenocorticotropic hormone (ACTH), catecholamine, glucocorticoids and mineral corticoid, are synthesized and secreted in greater abundance and more rapidly [16]. However, high levels of these hormones would have negative feedback on gonadotropin-releasing hormone (GnRH), and thus, the hypothalamic–pituitary–gonadal axis would be inhibited [17]. In addition, glucocorticoids could directly inhibit the release of estrogen and progesterone secreted by the ovary [18–20]. Notably, the inhibition of gonadotropins and lower estrogen concentrations in the plasma were associated with elevated serum lipid profiles [21]. Furthermore, apart from the activation of the HPA axis, another major metabolic change caused by trauma is a decrease in the normal metabolism of insulin, which means the occurrence of insulin resistance [22–24]. Insulin resistance has been reported to be associated with abnormal lipid metabolism [25]. Additionally, a rise in glucocorticoids has recently been suggested to be involved in insulin resistance and lipid abnormalities [26]. In this study, our findings showed that the serum levels of TG and LDL both increased, and the concentration of HDL decreased after surgical trauma. A possible explanation for the elevated lipid profiles might be that the stress response to surgical trauma, which may lead to the activation of the HPA axis and a rise in glucocorticoids, results in an increase in serum lipid profiles. Additionally, the activation of the HPA axis itself may inhibit the function of the ovary and then decrease estrogen, resulting in higher lipid profiles.

Apart from the neuroendocrine changes caused by the stress response to surgical trauma, surgery itself might also have effects on serum lipid levels. Huang et al. [27] investigated the serum lipid profiles of 600 patients with lung cancer before and after surgical treatment. Their findings showed that the levels of TC and LDL both increased after surgery (*p* < 0.05), and the levels of TG and HDL were also increased, although no statistically significant differences were observed. Similarly, our results showed that TG and LDL increased and HDL decreased after surgery. Huang et al. [27] noted that the increase in lipid profiles might be attributed to the fact that the tumor burden was reduced and the functions of organs were improved after surgery; thus, lipid synthesis might increase and consumption might decrease accordingly. As reported, lipids, especially cholesterol, play a vital role in cellular structure and function [28]. Tumor cells grow rapidly and consume many lipids, which results in lower lipid profiles in cancer patients [29–31]. However, the consumption of lipids is reduced to some extent if the tumor is removed; thus, the lipid profiles become elevated. Furthermore, strong evidence has indicated that the levels of TC are negatively correlated with the presence, development or metastasis of breast cancer [32–34]. However, the specific mechanisms between lipid profiles and breast cancer are unclear, so further studies are urgently warranted.

In this study, we found that 27.76% of patients with preoperative ortholiposis developed dyslipidemia after surgery. Jianxing et al. [35] reported that dyslipidemia was positively correlated with BMI. Similarly, our results also showed that patients with BMI > 24 had a higher risk of developing dyslipidemia after surgery. In terms of menopausal status, Croce et al. [36] reported that compared with premenopausal women, postmenopausal women had a higher risk of developing dyslipidemia. In accordance with their findings, we also found that postmenopausal patients had a higher risk of developing dyslipidemia than premenopausal patients after surgery.

Du et al. [37] reported that serum lipid profiles increased significantly after hysterectomy, and the changes were more remarkable in younger women. Similarly, Tian et al. [38] also reported that the younger group showed a greater increase in TC and LDL levels during chemotherapy than the 41–65-year-old group. Consistent with their findings, in our study, it is worth noting that the magnitude of the increased TG, TC and LDL was greater in the premenopausal group than in the postmenopausal group. A possible explanation may be that the neuroendocrine changes caused by the stress response to surgical trauma had more effects on the well-functioning ovaries of premenopausal women [37]. Moreover, it is not difficult to understand that postmenopausal ovarian function is poor; thus, external interference has limited effects on ovarian function.

Furthermore, we found that patients with preoperative dyslipidemia had a higher risk of increased lipid profiles after surgery than those with normal preoperative lipid levels. The reason may be that patients with dyslipidemia may have disorders of lipid metabolism, which makes them more likely to have lipid changes. Additionally, we found that patients with BC with PR positivity had a higher risk of increased lipid profiles after surgery than those with PR negativity. Unfortunately, very few studies have focused on the relationship between PR status and lipid metabolism, and more research should be carried out in the future to confirm this finding.

To the best of our knowledge, the present study was one of the studies with a relatively large sample to provide comprehensive information on the serum lipid profiles pre- and postoperation in both premenopausal and postmenopausal patients with BC. Moreover, factors potentially implicated in elevated lipid profiles after surgery were also identified. However, there are still several limitations that should be highlighted. Notably, the physical activities of the patients were largely reduced, and their diets tended to be high-fat or high-protein during the perioperative period, which might also enhance the elevated lipid profiles. Additionally, there is a lack of data on calorie intake and consumption, energy variation, body composition, axillary surgery procedures, the number of lymph nodes removed and surgery time. The exact mechanism of lipid increase is not yet clear, and many factors may also have intertwining effects on lipids. Last but not the least, as Li et al. [39] reported, preoperative lower TG and HDL-C level were risk factors of breast cancer patients. It is urgent for us to explore the relationship between dyslipidemia and CVD and the long-term prognosis of patients with BC.

## Conclusions

In summary, the serum lipid profiles of patients with BC may increase after surgery, especially in those who are premenopausal. Additionally, quite a few patients developed dyslipidemia after surgery, and postmenopausal and overweight patients may have a higher risk of being diagnosed with dyslipidemia. However, different surgical procedures did not show different effects on lipid profiles. Therefore, lipid monitoring, dyslipidemia prevention and corresponding interventions should be taken into consideration during the perioperative period.

## Data Availability

Data are available via direct requests to the corresponding author at West China Hospital and with permission from the Institutional Review Board and Ethics Committee of West China Hospital.

## Abbreviations

BC: Breast cancer
TG: Triglycerides
TC: Total cholesterols
LDL: Low-density lipoprotein
HDL: High-density lipoprotein
BMI: Body mass index
CVD: Cardiovascular disease
ECOG: Eastern Cooperative Oncology Group
SD: Standard deviation
ACTH: Adrenocorticotropic hormone
HPA: Hypothalamic–pituitary–adrenal
SNS: Sympathetic nervous system
GnRH: Gonadotropin-releasing hormone
ER: Estrogen receptor
PR: Progesterone receptor
TNM: Tumor Node Metastasis
SPSS: Statistical Package for the Social Sciences
